# TGF-β1 Promotes the Recovery of Dorsal Root Ganglion Neurons from Cisplatin-Induced Injury Through Smad4-Dependent Mechanism

**DOI:** 10.3390/cimb48040344

**Published:** 2026-03-25

**Authors:** Pan Wu, Yiling Wei, Xiang Chen, Qingmei Mo, Ming Zhuo

**Affiliations:** Medical School, Guizhou University, Guiyang 550000, China; gs.panwu23@gzu.edu.cn (P.W.); yilingweiv@163.com (Y.W.); xiangchen18@163.com (X.C.); qingmeimo@163.com (Q.M.)

**Keywords:** chemotherapy-induced peripheral neuropathy, dorsal root ganglion neurons, cisplatin, transforming growth factor-β, nerve injury recovery, suppressor of mothers against decapentaplegic 4

## Abstract

Chemotherapy-induced peripheral (CIPN) neuropathy is a common dose-limiting side effect affecting roughly 30–40% patients. Dorsal root ganglia (DRG) neurons are one of the main targets of CIPN as chemotherapy drugs may accumulate in DRG neurons. Chemotherapy drugs may induce direct damages on DRG neurons while also activating immune pathways, which results in the releasing of pro-inflammatory cytokines. This cascade may also damage neurons and amplify pain signaling. Transforming growth factor-β1 (TGF-β1) is a multifunctional cytokine with prominent immunomodulatory roles. Here, we report that TGF-β1 can promote axonal regeneration on DRG neurons injured by cisplatin via a suppressor of mothers against decapentaplegic (Smad) signaling pathway. To confirm the involvement of canonical TGF-β signaling, we applied the selective TGF-β type I receptor antagonist SB-431542 and performed a gene knockdown of Smad3 and Smad4, assessing their impacts on TGF-β1’s effects. Our results demonstrate that TGF-β1 could significantly enhance axonal regeneration in DRG, largely through a Smad4-dependent pathway, and we propose TGF-β1/Smad4 as a promising molecular target for treating CIPN.

## 1. Introduction

Chemotherapy-induced peripheral neuropathy (CIPN) is a frequent challenge for cancer treatment. Approximately one-third of patients receiving chemotherapy drugs would develop peripheral neuropathy of varying severity [1]. Cisplatin, a widely used platinum-based chemotherapy drug, would typically induce detectable peripheral neuropathy at a rate as high as 92% of patients. The symptoms include loss of vibration sense, proprioceptive deficits (ataxia), and deep tendon reflex loss, reflecting predominantly sensory neuronopathy [2]. CIPN can severely diminish quality of life, causing difficulties with fine motor tasks and ambulation; in extreme cases, patients may become functionally dependent due to sensory loss or intractable pain. Furthermore, CIPN often necessitates chemotherapy dose reduction or discontinuation, compromising optimal cancer treatment. Given its prevalence and impact, there is a pressing need to develop effective preventive therapies.

The primary site of damage in CIPN is the dorsal root ganglion (DRG), which houses the cell bodies of primary sensory neurons. Unlike the CNS, the DRG capillaries have incomplete tight junctions and high permeability, making DRG neurons especially vulnerable to circulating neurotoxic agents [3]. Consequently, drugs like cisplatin preferentially accumulate in the DRG and induce direct neuronal injury [4]. Cisplatin’s cytotoxic mechanism in neurons is mainly from its ability to form platinum–DNA crosslinks on both nuclear and mitochondria DNA, causing DNA crosslinks that block DNA replication and transcription. DNA damage in DRG neurons triggers cell stress responses and impairs the transcription of genes needed for neuronal function. Thus, cisplatin-treated neurons show depleted mtDNA, reduced oxidative phosphorylation, and excessive reactive oxygen species (ROS) generation [5]. Elevated ROS in turn damages cellular components (lipids, proteins, enzymes) and activates apoptosis pathways [6].

Transforming growth factor-β 1 (TGF-β1) is a cytokine that has garnered attention in the context of neural injury and repair because of its broad regulatory actions. TGF-β1 belongs to the TGF-β superfamily and is ubiquitously expressed in many tissues. It is best known as a master regulator of immune responses and tissue healing [7]. In the nervous system, TGF-β1 is one of the most potent immunosuppressive cytokines, capable of shifting immune cells toward anti-inflammatory phenotypes and suppressing the release of pro-inflammatory mediators [8]. Immunohistochemical studies show that TGF-β1 is abundantly present in the microenvironment of the nerve bridge (the tissue that connects the proximal and distal stumps after nerve transection). Importantly, the TGF-β1-treated rats showed reduced expression of pro-inflammatory cytokines like IL-1β in the DRG and nerve and exhibited less pain behavior than untreated injured controls. This indicates that TGF-β1 can simultaneously stimulate neuronal regeneration and mitigate neuropathic pain, likely by creating a more favorable immunological environment [9,10]. In cultured DRG neurons, addition of TGF-β1 significantly increases the proportion of neurons that extend neurites and the average neurite length, comparable to the effects of nerve growth factor (NGF) in some studies. The underlying mechanisms involve changes in gene expression: the TGF-β1 treatment of neuron was found to upregulate brain-derived neurotrophic factor (BDNF) and its receptor TrkB, indicating that TGF-β1 may boost the autocrine neurotrophic support within neurons [11,12]. However, its underlying mechanisms have not yet been fully elucidated.

Mechanism-wise, TGF-β1 activates the Smad (suppressor of mothers against decapentaplegic) signaling pathway in neurons by specifically phosphorylating Smad2/3, which then form complexes with Smad4 and are then translocated to the nucleus to drive the transcription of regeneration-associated genes [13]. In the canonical TGF-β pathway, Smad2/3 are the R-Smads, while Smad4 serves as the essential co-mediator. Receptor-activated Smad2/3 bind Smad4 to form heteromeric transcriptional complexes that translocate to the nucleus and bind DNA [14]. Many TGF-β-dependent effects require Smad4-mediated transcriptional activation [15]. Notably, in models of peripheral nerve injury, Smad4 has been specifically linked to enhanced regeneration: one recent review reported that the activation of Smad1 and Smad4 promotes nerve regrowth [16].

Given the complex, multi-factorial nature of CIPN, we propose that TGF-β1 could be an ideal therapeutic candidate for CIPN as it inherently possesses anti-inflammatory, antioxidant, and neurotrophic properties. By elucidating how TGF-β1 impacts these factors and pathways, we seek to clarify the molecular mechanisms through which TGF-β1 might mitigate CIPN and provide a foundation for future multi-modal therapeutic strategies.

## 2. Methods

### 2.1. Animals

Specific pathogen-free (SPF) grade 8–12 weeks old C57BL/6 mice (purchased from Chongqing Tengxin Biotechnology Co., Ltd., Chongqing, China) were used. Mice were housed in a barrier environment with ≤5 mice per cage, maintained on a 12 h light/12 h dark cycle, and the ambient temperature was controlled at 20–24 °C. Mice were fed ad libitum with standard rodent chow and sterilized chow. Standard rodent chow and sterilized drinking water were ingested ad libitum. The study was conducted in accordance with ARRIVE guidelines. All animal experiments followed the requirements of the Guizhou University Ethics Committee (Grant No. EAE-GZU-2022-T035).

### 2.2. Isolation, Culture, and Processing of Primary Dorsal Root Ganglion Neurons

Dorsal root ganglion neurons were isolated following the described protocol with some modifications [17,18,19]. Briefly, ganglia were removed from all spinal cord segments, placed in ice-cold dissection solution (130 mM NaCl, 5 mM KCl, 2 mM KH_2_PO_4_, 1.5 mM CaCl_2_, 6 mM MgCl_2_, 10 mM glucose, and 10 mM Hepes, pH 7.2. All from Solarbio, Beijing, China), and connective tissues were trimmed. The ganglia were incubated with 1 μg/mL collagenase A and 1 mg/mL trypsin for 1 h at 37 °C, washed, incubated with 50 μg/mL DNAzyme I (both from Solarbio, Beijing, China), dissociated by 20 passes through a 70 μm filter by gentle grinding, and centrifuged at 1200 rpm for 3 min to precipitate the ganglia [20]. The precipitate was washed with DPBS, centrifuged and resuspended in DMEM/F12 (BasalMedia, Shanghai, China) supplemented with 10% FBS and 1% penicillin/streptomycin (both from Biosharp, Beijing, China). Neuronal cells were inoculated at 3 × 10^3^ on 24-well or 6-well plates with coverslips pre-coated in 10 μg/mL laminin and 100 μg/mL poly-L-ornithine (both from Sigma-Aldrich, St. Louis, MO, USA), respectively. The plates were then incubated in a 37 °C incubator containing 5% CO_2_. Nerve damage was induced using 12 μM cisplatin (Merck, HE, Darmstadt, Germany). After 24 h of treatment, cisplatin was removed by replacement with fresh medium. NGF, TGF-β1, and IL-2 were prepared from 10 μg/mL stock solution prepared in PBS, different concentrations of cytokines were added, and the cells were incubated for another 48 h.

### 2.3. Tissue Collection and Processing

Mice were randomly assigned to five experimental groups (*n* = 4 per group). Cisplatin injury models are: in the cisplatin-only group, mice were administered cisplatin (6 mg/kg) intraperitoneally (i. p.) once daily on days 1–5; in the cisplatin + saline group, mice were administered the same cisplatin regimen (6 mg/kg, i. p., days 1–5) followed by sterile saline (i. p.) on days 6 and 8; in the cisplatin + TGF-β1 group, mice were administered cisplatin (6 mg/kg, i. p., days 1–5) followed by recombinant TGF-β1 (0.1 mg/kg, i. p.) on days 6 and 8; in the cisplatin + TGF-β1 + SB431542 group, mice were administered cisplatin (6 mg/kg, i. p., days 1–5) followed by co-administration of TGF-β1 (0.1 mg/kg, i. p.) and SB431542 (10 mg/kg, i. p.) on days 6 and 8; in the control group, mice were administered sterile saline (i. p.) on days 1–5 and again on days 6 and 8. All injections were administered via the intraperitoneal route, and mice were euthanized by cervical dislocation at the study endpoint. Immediately after sacrifice, bilateral hind paws (including the distal glabrous footpads) were collected. These samples were fixed overnight at room temperature in 4% paraformaldehyde (PFA). After fixation, tissues were rinsed in phosphate-buffered saline (PBS) and decalcified in 10% EDTA (pH 7.4) at 4 °C until bone was softened enough. Cryoprotected tissues were embedded in optimal cutting temperature (OCT) compound and frozen. Serial longitudinal sections of the hind paw (10 μm thickness) were cut on a cryostat (−20 °C) and mounted onto glass slides.

### 2.4. Immunofluorescence Staining, Image Acquisition, Neurite Length Measurement and Immunofluorescence Staining Intensity Analysis

DRG neurons were inoculated onto glass cell culture coverslips for immunofluorescence staining. After treatment, the cells were fixed in 4% paraformaldehyde (Biosharp, Beijing, China), permeabilized with PBS solution containing 0.1% Triton X-100 (Solarbio, Beijing, China), and blocked with 3% bovine serum albumin (BSA) (Biofroxx, SN, Einhausen, Germany) in PBS. The primary antibodies used were: rabbit anti-Neurofilament 200 (NF 200) (N4142, 1:50,000, Merck, HE, Germany), Iba1/AIF-1 (E404W) XP^®^ Rabbit mab (#17198, Cell Signaling TECHNOLOGY^TM^, Danvers, MA, USA), rabbit anti-PGP9.5 (Clone number: JM10-59, ET1703-22, 1:500, Hangzhou Huaan Biotechnology Co., Ltd., Hangzhou, China), Smad3 (sc-133098), Smad4 (sc-7966), which were both from Santa Cruz, and, after washing the slides three times with PBS (PBST) containing 0.1% Tween, the slides were incubated with the secondary antibody (Goat Anti-Mouse IgG/APC purchased from Solepol Science and Technology Co., Ltd. No. K0031G-APC, Beijing, China, Goat anti-Mouse IgG (H + L) Cross-Adsorbed Secondary Antibody, Alexa Fluor™ 488 (A-11001), or cy 5^TM^ goat anti-mouse IgG (H + L) (A10524), Invitrogen, both 1:2000, Carlsbad, CA, USA) for 30 min of incubation. After washing three more times with PBST, the slices were blocked with ProLong™ Gold antifade reagent with DAPI (P36931, Invitrogen, CA, USA) and imaged using an Olympus fluorescence microscope (IX 73, OLYMPUS cellSens Standard 4.1.1) using a 40× objective lens. To quantify the length of DRG neurite extensions, cell bodies and neurites of DRG neurons were visualized using neuron-specific cytochrome protein NF 200 staining. Neurites were traced using the Segmented Lines tool in the NIH ImageJ software (ImageJ 1.54f) and their lengths were analyzed. For each experimental condition, at least 10 randomly selected DRG neurons were analyzed. For each DRG cell with multiple neurites, 3–4 neurites were traced, and the longest length from the cell body to the tip of the neurite was measured. When a neurite branched, the tracing continued along the branch that led to the most distal tip. We measured relatively long neurites and selected the longest ones for statistical analysis. The average length of these neuromasts was calculated. Three independent biological replicates were performed. Data are expressed as mean ± SEM, derived from more than 50 neurites analyzed for each experimental condition. Fluorescence intensity of individual DRG neurons was measured using ImageJ software. Cell bodies or neuromasts were selected using the Freehand tool in the NIH ImageJ software (ImageJ 1.54f) and the average intensity was measured. For each treatment condition, immunofluorescence intensities from at least 15 DRG neurons were analyzed. Three independent biological replicates were performed and the mean ± SEM values of at least 45 DRG neurons for each condition were calculated.

### 2.5. Real-Time Quantitative Polymerase Chain Reaction

Total RNA was extracted from DRG neurons by the SteadyPure Rapid RNA Extraction Kit (Accurate Biology, Changsha, China) and reverse transcribed into cDNA using Evo M-MLV Reverse Transcription Premixed Kit (Accurate Biology, Changsha, China). Real-time PCR was performed using a CFX 96 real-time system (Biorad, Hercules, CA, USA). Real-time quantitative PCR was performed using the SYBR Green Pro Taq HS qPCR Premix Kit, purchased from Accurate Biotechnology Co., Ltd., Changsha, China. The internal housekeeping genes used for synthesis were β-2-microglobulin (B2 M) and 18S ribosomal RNA (18s rRNA), and the relative mRNA amount was calculated using the 2^−ΔΔCt^ method [21]. All qPCR primers were synthesized by Sangon Biotech Co (Shanghai, China). The forward and reverse sequences of each set of gene-specific primers are shown in Table 1.

### 2.6. Gene Knockdown and Use of Transforming Growth Factor-β Blockers

For RNA interference, all small interfering RNAs (siRNAs) were synthesized by Genepharma (Shanghai, China). siRNA sequence design is shown in Table 2. The siRNAs were transfected into DRG neurons using the RNAiMAX reagent (Invitrogen, CA, USA) according to the reagent instructions. After pretreatment with siRNA in neuronal basal medium for 24 h, the siRNA-containing medium was removed and co-cultured for 48 h with a complete medium with NGF or TGF-β1 instead. The TGF-β receptor kinase inhibitor SB-431542 (Catalog No.: HY-10431, MedChemExpress, Monmouth Junction, NJ, USA) was dissolved in DMSO to make a masterbatch concentration of 10 mM and was always used at a final concentration of 2 μM.

### 2.7. Statistical Analysis

To ensure the reproducibility and stability of the experimental results, all experiments in this study were performed as at least three independent biological replicates. Experimental data were expressed as mean ± standard error (mean ± SEM) and statistical analysis was completed by GraphPad Prism 9.5 software. Specifically, for the initial assessment of differences between multiple groups, a one-way ANOVA was used to identify overall differences between experimental groups. Subsequently, for the comparison of the two groups of data, an independent *t*-test was applied to determine the significance of the differences in means between the groups. The significance level was set at *p* < 0.05 as the threshold for statistical significance, and differences between groups were considered significant only when the *p* value was less than 0.05. All statistical tests were two-sided. A one-way Kruskal–Wallis test was performed using nonparametric analysis, followed by a post hoc Dunn’s test for secondary data verification.

## 3. Results

### 3.1. TGF-β1 Promotes the Growth of Neurite in DRG Neurons Cultured In Vitro

**Figure 1:**
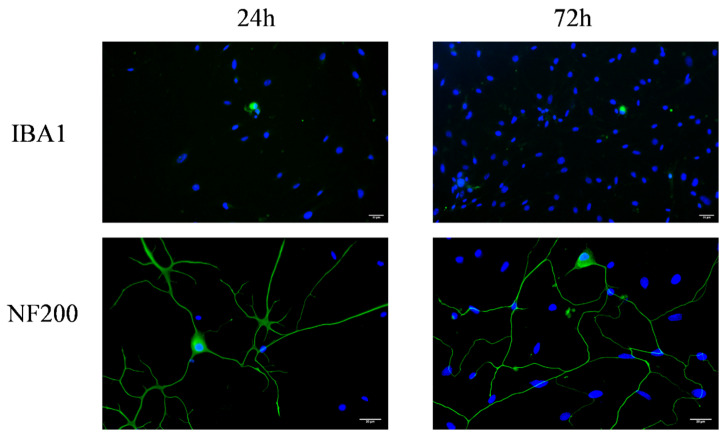
The proportion of glial cells and macrophages in cultured DRG cells. DRG neurons were cultured in full medium for 24 h or 72 h. Cells were visualized by immunofluorescence staining with NF 200 or IBA 1 (green) and cell nuclei were visualized by DAPI (blue). Scale bar = 20 μm.

There are many types of cells that may respond to TGF-β1, including microglia cells and macrophages. In our primary DRG culture, they may also exist. To better evaluate their number in our culture, we performed immunofluorescence staining on primary mouse DRG cultures after 24 or 72 h in vitro. This analysis revealed that the majority of cells were NF200-positive DRG neurons, whereas Iba1-positive glial cells and macrophages accounted for only a negligible fraction of the total cell population (Figure 1).

**Figure 2:**
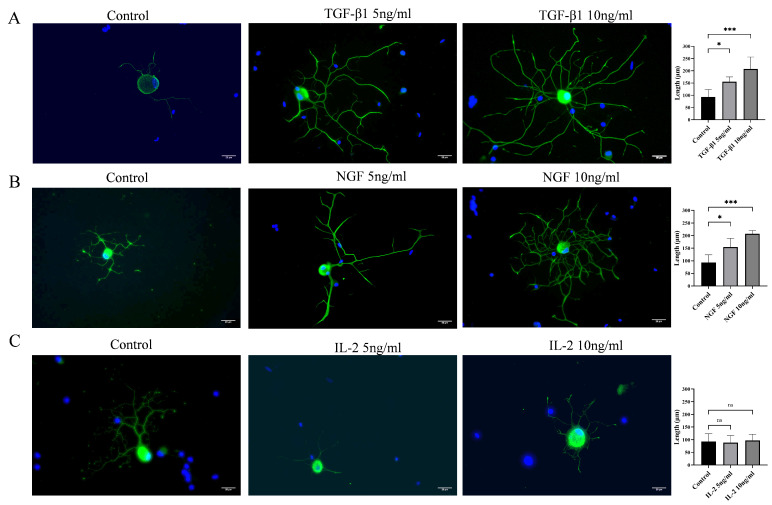
TGF-β1 promotes the growth of neurite on DRG neurons cultured in vitro. DRG neurons were cultured in neurobasal medium containing different concentrations of TGF-β1/NGF/IL-2 for 72 h. Cells were visualized by immunofluorescence staining with NF 200 (green) and cell nuclei were visualized by DAPI (blue). Scale bar = 20 μm. The neurite length of DRG neuron was quantified. (**A**) DRG neurons cultured with TGF-β1; (**B**) DRG neurons cultured with NGF; (**C**) DRG neurons cultured with IL-2. Data are expressed as mean ± SEM (*n* = 4 per group). * *p* < 0.05, *** *p* < 0.001, ns: not significant.

To assess the effect of cytokines on the growth of protrusions in DRG neurons, the length of protrusions from the control group and from the group treated with different concentrations of cytokines was measured. As shown in Figure 1, DRG neurons treated with NGF and TGF-β1 promoted the growth of neurites in DRG neurons (Figure 2A,B), whereas IL-2, another widely used cytokine, did not show any effect on the growth of neurites after 24 h of treatment (Figure 2C).

### 3.2. TGF-β1 Promotes Neurite Regeneration in Cisplatin-Injured DRG Neurons

**Figure 3:**
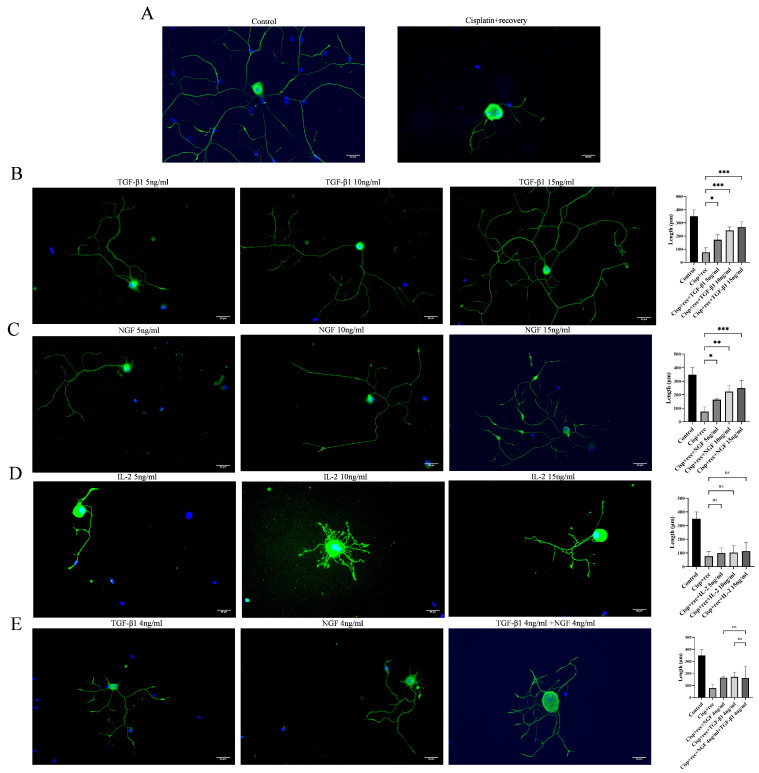
TGF-β1 promotes axonal regeneration in DRG neurons after cisplatin injury. DRG neurons were treated with 12 μM cisplatin for 24 h and then cultured in full medium supplemented with different concentrations of TGF-β1/NGF/IL-2 for 48 h. Cells were visualized by immunofluorescence staining with NF 200 (green) and cell nuclei were visualized by DAPI (blue). Scale bar = 20 μm. The neurite length of DRG neuron was quantified. (**A**) DRG neurons with or without cisplatin injury; (**B**) DRG neurons cultured in NGF-containing medium; (**C**) DRG neurons cultured in TGF-β1-containing medium; (**D**) DRG neurons cultured in IL-2-containing medium; (**E**) DRG neurons cultured in NGF- and TGF-β1-containing medium. Data are expressed as mean ± SEM (*n* = 4 per group), * *p* < 0.05, ** *p* < 0.01, *** *p* < 0.001, ns: not significant.

**Figure 4:**
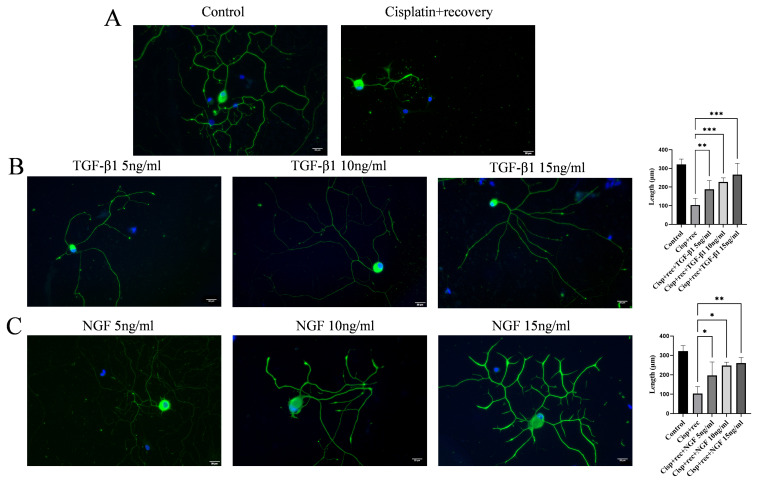
TGF-β1 promotes axonal regeneration in DRG neurons after 8 μM cisplatin injury. DRG neurons were treated with 8 μM cisplatin for 24 h and then cultured in full medium supplemented with different concentrations of TGF-β1/NGF for 48 h. Cells were visualized by immunofluorescence staining with NF 200 (green) and cell nuclei were visualized by DAPI (blue). Scale bar = 20 μm. The neurite length of DRG neuron was quantified. (**A**–**C**) are the same as in Figure 3 (*n* = 4 per group), * *p* < 0.05, ** *p* < 0.01, *** *p* < 0.001.

To investigate the effect of TGF-β1 on axonal regeneration in cisplatin-injured DRG neurons, we treated cisplatin-injured DRG neurons with different concentrations of TGF-β1 in Figure 3 and Figure 4. Immunofluorescence staining showed that TGF-β1 significantly promoted axonal regeneration in a dose-dependent manner (Figure 3B), and its effect was similar to that of NGF (Figure 3C). Parallel experiments using IL-2 showed that IL-2 could not promote synapse regeneration in DRG neurons after cisplatin injury (Figure 3D). We further tested the combination of NGF and TGF-β1, as shown in Figure 3E. This combination did not show additive or synergistic effects on the axonal regeneration of DRG neurons after injury.

### 3.3. SB431542 Inhibits the TGF-β1-Induced Growth of DRG Neurites and Blocks Smad4 Nuclear Translocation

**Figure 5:**
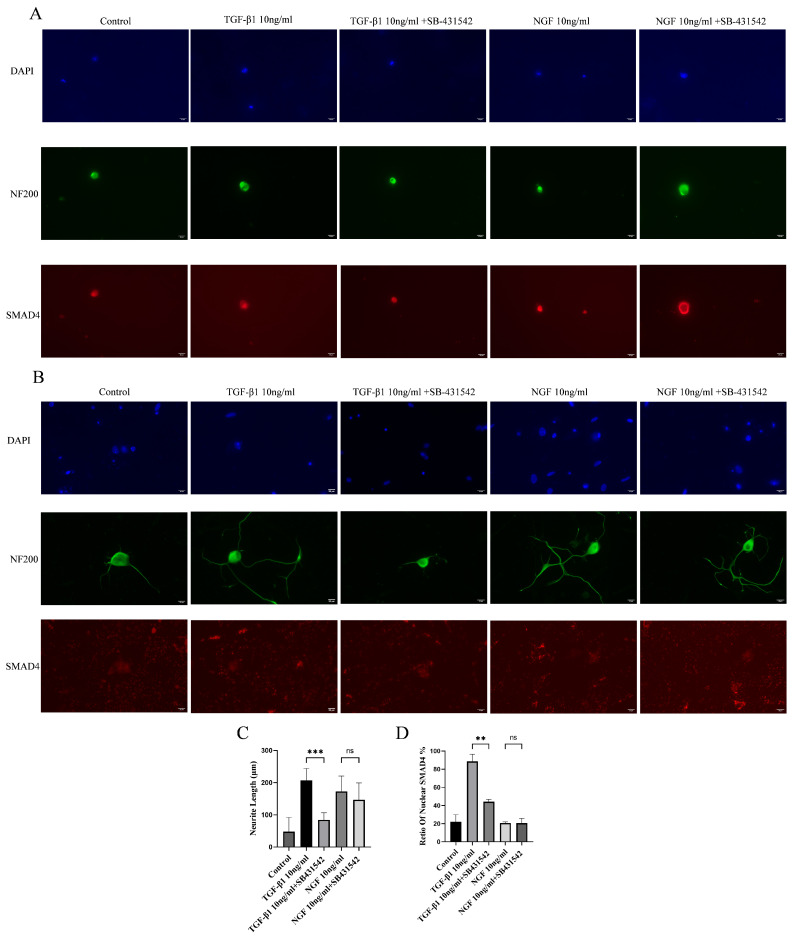
SB431542 inhibits the TGF-β1-induced neurite growth in DRG neuron and blocks Smad4 nuclear translocation. DRG neurons were treated with SB431542 and TGF-β1 for 72 h. Changes in the length of protrusions of DRG neurons were observed after 24 or 72 h of control and TGF-β1/NGF-combined SB-431542 treatment. Cells were visualized by immunofluorescence staining with NF 200 (green) and cell nuclei were visualized by DAPI (blue). Smad4 was visualized by immunofluorescence staining (red). Scale bar = 20 μm. The neurite length of DRG neuron was quantified. Neurite outgrowth was evaluated at 72 h, whereas Smad nuclear translocation was assessed at 24 h to capture the signaling event. (**A**) The TGF-β1 + SB-431542 group received TGF-β1 and SB-431542, the NGF + SB-431542 group received NGF and SB-431542; (**B**) the same as (**A**); (**C**) bar graph of average neurite length for 72 h; (**D**) bar graph showing Smad4 nuclear translocation ratio for 24 h; data are expressed as means ± SEM (*n* = 4 per group), ** *p* < 0.01, *** *p* < 0.001, ns: not significant.

SB431542 is a small-molecule kinase inhibitor that specifically inhibits the type I TGF-β1 receptor (ALK5), thereby blocking the phosphorylation and nuclear translocation of Smad2/3, and in turn suppressing TGF-β1-mediated downstream transcriptional activation. Therefore, we tested on DRG neurons if SB-431542 could block the role of TGF-β1. NF 200 staining showed that DRG neurons in the SB-431542 + NGF group could display normal axon growth, and in the SB-431542 + TGF-β1 group, axon growth was completely inhibited, while in the SB-431542 + TGF-β1 group, axon growth was completely inhibited (Figure 5A). Similarly, DRG neurons in the TGF-β1 group could show Smad4 nuclear translocation (Figure 5B). These results suggest that SB-431542 inhibits Smad pathway activation blocking TGF-β1 and induces Smad4 nuclear translocation.

### 3.4. Smad4 siRNA Suppresses TGF-β1-Triggered DRG Neurite Outgrowth and Inhibits Smad4 Nuclear Translocation

**Figure 6:**
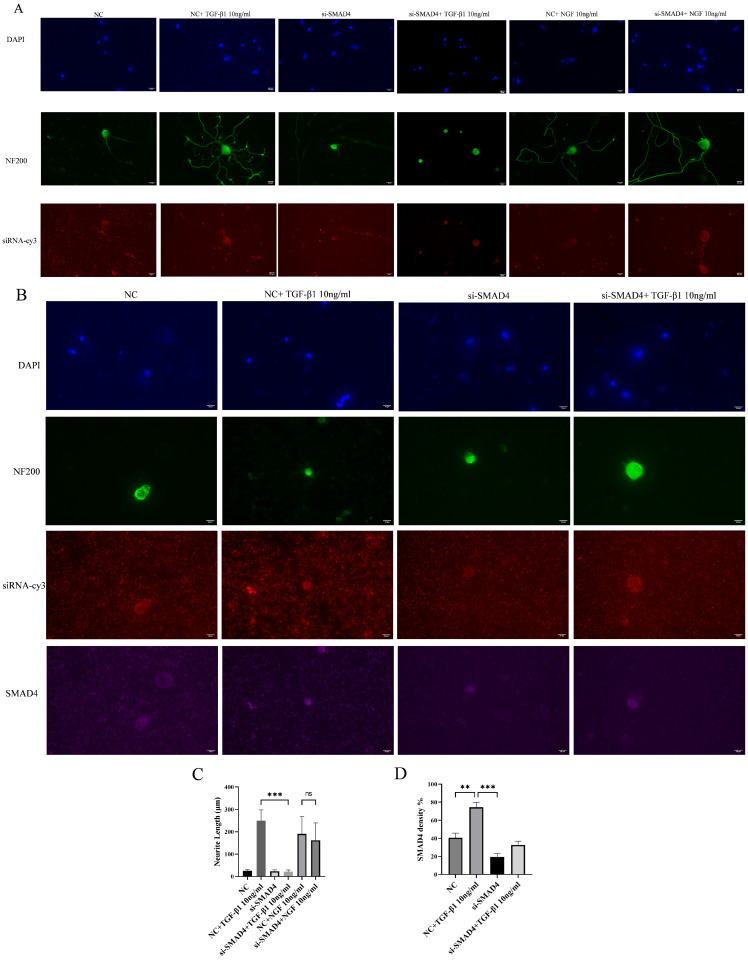
Smad4 siRNA suppresses TGF-β1-triggered DRG neurite outgrowth and inhibits Smad4 nuclear translocation. Changes in the length of protrusions of DRG neurons were observed after 24 or 72 h of control and TGF-β1/NGF-combined siRNA transfection. Cell bodies and neurites were visible by immunofluorescence staining with NF 200 (green) and labeled with cy 3 for si-Smad4, APC for Smad4. Scale bar = 20 μm. The neurite length of DRG neuron was quantified. Neurite outgrowth was evaluated at 72 h, whereas Smad nuclear translocation was assessed at 24 h to capture the signaling event. (**A**) The NC group received non-specific siRNA, the NC + TGF-β1 group received non-specific siRNA and TGF-β1, the si-Smad4 group received Smad4 siRNA, si-Smad4 + NGF group received Smad4 siRNA and NGF, and si-Smad4 + TGF-β1 group received Smad4 siRNA and TGF-β1; (**B**) the same as (**A**); (**C**) bar graph of average neurite length for 72 h; (**D**) bar graph showing Smad4 density for 24 h; data are expressed as means ± SEM (*n* = 4 per group), ** *p* < 0.01,*** *p* < 0.001, ns: not significant.

In the Smad4 siRNA + NGF treatment group, DRG neurons showed normal neurite extension and cytoplasmic distributions of Smad4. However, in the Smad4 siRNA + TGF-β1 group, neurite extension was entirely inhibited, and Smad4 remained cytoplasmic without nuclear localization (Figure 6A,B). This suggests that Smad4 knockdown effectively prevents TGF-β1-induced neurite outgrowth and Smad4 nuclear translocation.

### 3.5. Smad3 siRNA Abrogates the TGF-β-Mediated Promotion of DRG Neurite Growth and Prevents Smad3 Nuclear Translocation

**Figure 7:**
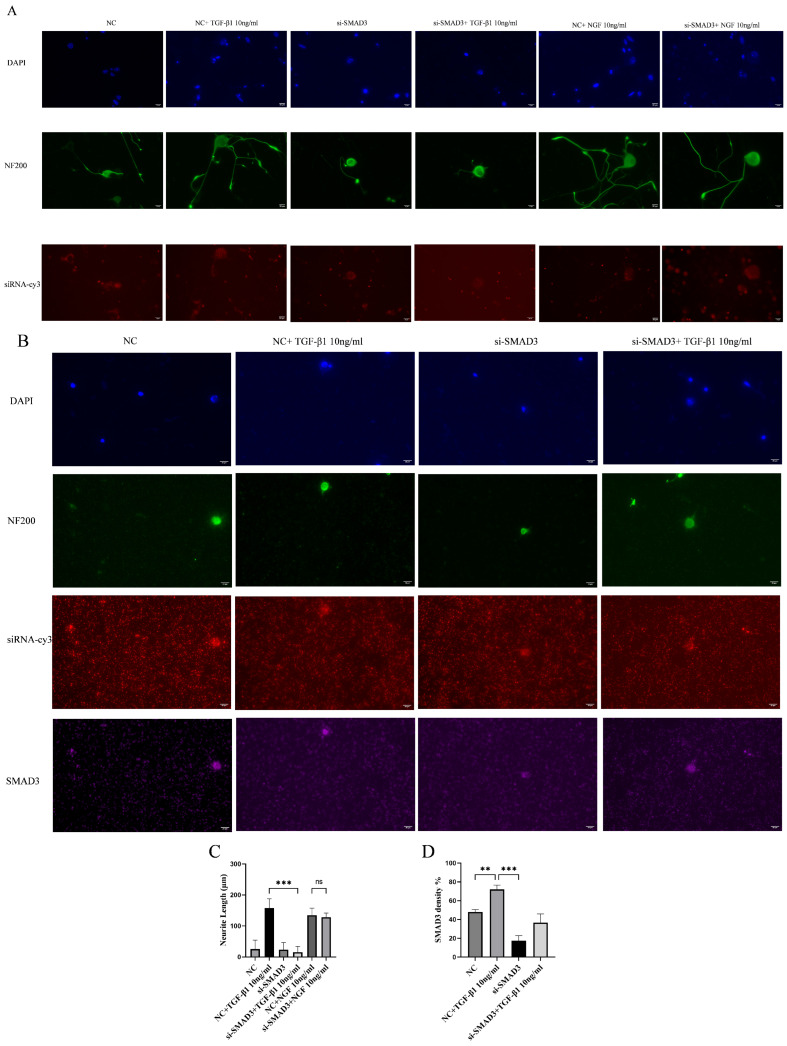
Smad3 siRNA abrogates the TGF-β1-mediated promotion of DRG neurite growth and prevents Smad3 nuclear translocation. Changes in the length of protrusions of DRG neurons were observed after 24 or 72 h of control and TGF-β1/NGF-combined siRNA transfection. Cell bodies and neurites were visible by immunofluorescence staining with NF 200 (green) and labeled with cy 3 for si-Smad3, APC for Smad4. Scale bar = 20 μm. The neurite length of DRG neuron was quantified. Neurite outgrowth was evaluated at 72 h, whereas Smad nuclear translocation was assessed at 24 h to capture the signaling event. (**A**) The NC group received non-specific siRNA, the NC + TGF-β1 group received non-specific siRNA and TGF-β1, the si-Smad3 group received Smad3 siRNA, si-Smad3 + NGF group received Smad3 siRNA and NGF, and si-Smad3 + TGF-β1 group received Smad3 siRNA and TGF-β1; (**B**) the same as (**A**); (**C**) bar graph of average neurite length for 72 h; (**D**) bar graph showing Smad3 density for 24 h; data are expressed as means ± SEM (*n* = 4 per group), ** *p* < 0.01,*** *p* < 0.001, ns: not significant.

NGF-treated DRG neurons displayed neurite extension and cytoplasmic Smad3 localization, as shown by NF200 staining and immunocytochemistry. In contrast, DRG neurons treated with Smad3 siRNA + TGF-β1 exhibited a complete absence of neurite outgrowth, and Smad3 signal remained confined to the cytoplasm with no evidence of nuclear translocation (Figure 7A,B). These observations indicate that Smad3 siRNA suppresses TGF-β1-mediated promotion of neurite outgrowth and blocks Smad3 nuclear translocation.

### 3.6. TGF-β1 Attenuates the Reduction in Nerve Fiber Density Caused by Cisplatin Injury

**Figure 8:**
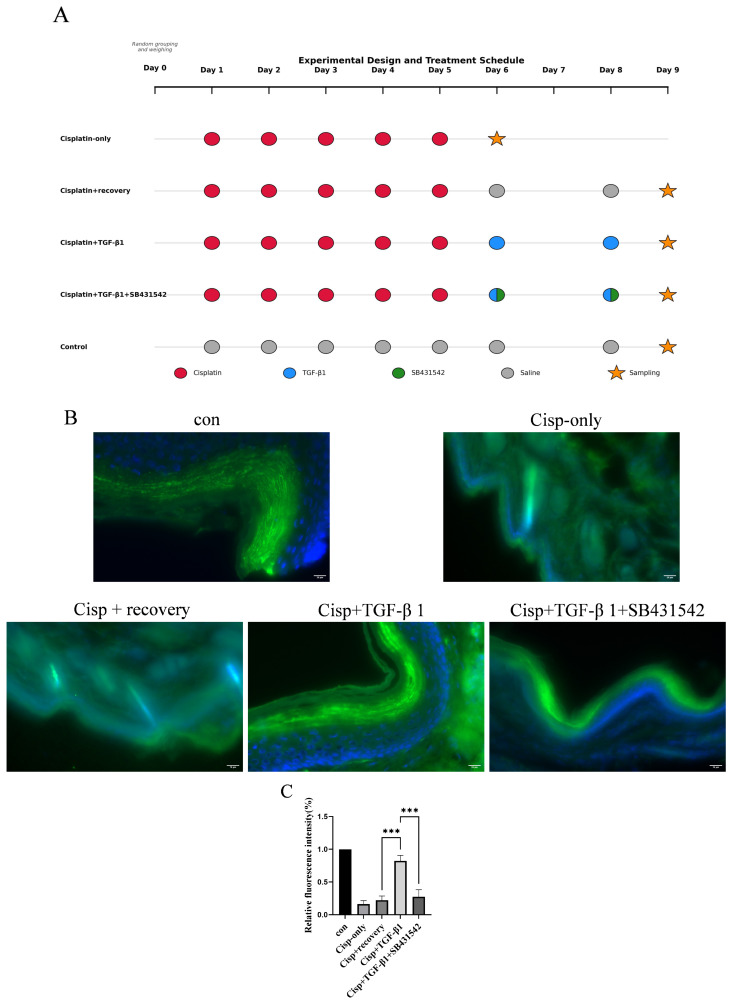
TGF-β1 attenuates the reduction in nerve fiber density caused by cisplatin injury. (**A**) Experimental design timeline. (**B**) Nerve fibers were visualized by immunofluorescence staining with PGP 9.5 (green) and cell nuclei were visualized by DAPI (blue). Scale bar = 20 μm. (**C**) Relative fluorescence intensity (%). Data are expressed as means ± SEM (*n* = 4 per group), *** *p* < 0.001.

Cisplatin-induced peripheral neurotoxicity is known to cause the degeneration of cutaneous sensory fibers, and previous studies have documented a significant loss of PGP9.5-positive epidermal nerve fibers after cisplatin treatment [22]. In this study, PGP9.5 immunofluorescence was performed on hindpaw skin sections and nerve fiber density was quantified by image analysis. The control group exhibited the highest mean nerve fiber density. In contrast, both the cisplatin-only and cisplatin + saline groups showed a significant reduction in PGP9.5-labeled fiber density relative to control. Notably, the cisplatin + TGF-β1 group retained a substantially higher fiber density (not significantly different from control), indicating that exogenous TGF-β1 preserved nerve fibers. By contrast, inclusion of the TGF-β signaling inhibitor SB431542 in the cisplatin + TGF-β1 + SB431542 group abolished this protective effect: fiber density in this group was significantly lower and comparable to the cisplatin-only group (Figure 8B).

### 3.7. SB-431542 Acts on DRG Neurons via TGF-β-Mediated Signaling Pathway and Blocks TGF-β1-Induced Transcriptional Responses

**Figure 9:**
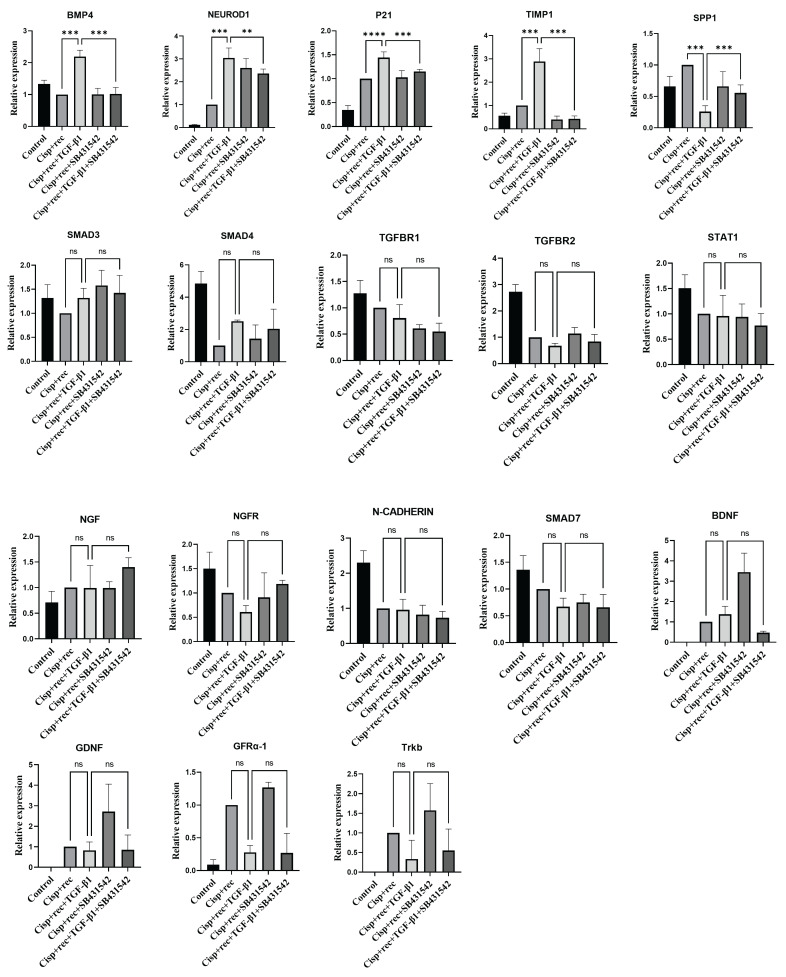
SB-431542 acts on DRG neurons through TGF-β1-mediated signaling pathway to block TGF-β1-induced transcriptional responses. RT-qPCR analyses of the expression of related genes in DRG neurons treated with TGF-β1 or TGF-β1 + SB2431542 after cisplatin injury. Data are presented as mean ± SEM (*n* = 5 per group). ** *p* < 0.01, *** *p* < 0.001, **** *p* < 0.0001, ns: not significant.

While finding that TGF-β is a major functional cytokine in DRG neurons, we further explored the downstream signaling molecules of TGF-β1 in DGR neurons. Their protein expression was detected. RT-qPCR results showed that the addition of TGF-β1 or SB431542 did not affect the expression of Smad3, Smad4, NGF, NGFR, TGFBR1, TGFBR2, STAT1, N-CADHERIN, SMAD7, BDNF. GDNF, TRKB, or GFRα1. SPP1 were significantly downregulated and BMP4, NEUROD1, P21, and TIMP1 were significantly upregulated when compared with the recovery group with the addition of TGF-β1. Compared to restoration of TGF-β1, SPP1 were significantly upregulated and P21, BMP4, NEUROD1, and TIMP1 were significantly downregulated with the addition of SB431542in Figure 9.

## 4. Discussion

In this work, we revealed that TGF-β1 treatment triggers the translocation of Smad2/3 and Smad4 from the cytoplasm to the nucleus in DRG neurons. This nuclear translocation was abolished by SB431542 or by siRNA-mediated knockdown of Smad3/Smad4, indicating that the pro-regenerative effect of TGF-β1 on DRG axons is contingent upon Smad pathway activation. We also considered whether a minor population of immune cells (e.g., macrophages or microglia) in the DRG might respond to TGF-β1 and contribute to neural regeneration. However, these cells were exceedingly scarce in DRG cultures, with no evidence of their clustering or association with DRG neurons. This finding largely rules out the possibility that TGF-β1 promotes regeneration indirectly via immune cells. Furthermore, qPCR analyses of key molecules in non-canonical (Smad-independent) TGF-β1 signaling pathways showed no significant changes in expression after TGF-β1 treatment, including for BMP4, NEUROD1, P21, TIMP1, and so on. Collectively, these results indicate that TGF-β1 promotes axonal regeneration primarily by directly activating the Smad3/Smad4 signaling axis in DRG neurons, rather than indirectly through other cells or via Smad-independent pathways.

The TGF-β signaling pathway plays a notable role in repairing cisplatin-induced nerve injury through both its canonical and non-canonical branches. As a multifunctional cytokine, TGF-β promotes peripheral nerve repair through multiple mechanisms. It regulates Schwann cell proliferation and phenotypic transformation, recruits immune cells for debris clearance and regeneration, enhances blood–nerve barrier function to stimulate axonal growth, and even helps prevent the premature myelination of regenerating axons [14]. Concurrently, TGF-β can also activate non-canonical pathways such as PI3K/Akt, ERK, and Rho-family GTPases signaling routes closely associated with neuronal survival and neurite outgrowth [23]. Smad2 and Smad3, the two receptor-regulated Smads (R-Smads) downstream of TGF-β, have distinct functions in nerve regeneration [16]. Smad3 activation has been shown to positively influence axonal regrowth. For example, in peripheral nerve injury models, Smad3 signaling promotes neuroglial bridging at the lesion site in part by upregulating regeneration-associated genes such as the connective tissue growth factor (CTGF). By contrast, recent studies indicate that while upregulation of Smad4 (but not Smad2) aids peripheral nerve regeneration, elevated Smad2 is associated with neurodegenerative changes. Our experiments confirmed that siRNA-mediated knockdown of Smad3 significantly reduced TGF-β-induced neurite outgrowth, indicating that Smad3 contributes to TGF-β’s axonal repair effects.

However, because Smad3 must form a complex with the common partner Smad4 to activate transcription, these results imply that Smad4 may be the key player. Studies have shown that Smad4 is necessary for the formation of stable transcriptional complexes: heterotrimeric assemblies containing Smad4 are more common and stable than other Smad oligomers, and Smad4 is required to recruit transcriptional co-activators such as p300/CBP for effective gene expression. Indeed, in vivo data demonstrate that loss of Smad4 severely impairs tissue repair, whereas Smad3-deficient animals often exhibit accelerated healing. Thus, our results suggest that while Smad3 is involved, the regenerative signaling of TGF-β in injured nerves depends primarily on the Smad4-containing complex. Smad3 readily partners with Smad4 to drive transcription of regeneration-related genes, such that inhibiting Smad3 disrupts TGF-β’s pro-regenerative signaling. In summary, TGF-β signaling plays a crucial role in repairing cisplatin-induced nerve injury, but its regenerative benefits are largely driven by Smad4-dependent pathways.

To further investigate TGF-β1’s mechanism of action, we examined changes in various downstream molecules. Key TGF-β signaling components (TGFBR1, TGFBR2, Smad3, Smad4, Smad7) showed no significant expression changes after TGF-β1 treatment. Similarly, immune-related genes like STAT1 remained unchanged. These findings suggest that TGF-β does not exert its effects by upregulating its own receptors or core Smad proteins in our model. However, several genes did change significantly with TGF-β treatment, including BMP4, NEUROD1, P21, SPP1, and TIMP1. These factors are closely associated with neuronal growth and regeneration: TGF-β upregulates BMP4, activating the Smad1/5/8 pathway and promoting sensory neuron axon growth. Exogenous BMP4 enhances DRG axon extension and branching, improving regenerative capacity [24]. NeuroD1, which regulates neuronal differentiation and axon growth during development, is upregulated by TGF-β. In the adult CNS, NeuroD1 overexpression enhances axonal regeneration and functional recovery [25]. TGF-β also upregulates p21^Cip1/Waf1^. While nuclear p21 causes cell cycle arrest, cytoplasmic p21 binds Rho kinase to promote neurite outgrowth. Applying exogenous cytoplasmic p21 to injury sites improves axon regeneration and motor function after spinal cord injury [26]. Conversely, TGF-β downregulates SPP1 (osteopontin, OPN), a factor produced by macrophages and Schwann cells, upregulated after peripheral nerve injury [27]. Recombinant OPN does not directly stimulate sensory axon growth in vitro, but OPN knockout mice show severely impaired sciatic nerve regeneration, highlighting OPN’s critical role in repair [28]. Finally, TGF-β upregulates TIMP1 to modulate the extracellular matrix. Moderate TIMP1 stabilizes the matrix, whereas excessive TIMP1 restricts axon extension, causing enlarged growth cones and shorter axons [29]. Thus, TIMP1 plays a dual role in regeneration, requiring a balance that protects tissues without inhibiting axonal regrowth.

Previous studies did not fully clarify the role of TGF-β1 in dorsal root ganglion (DRG) neurons. Early evidence suggested that the neurotrophic effects of TGF-β1 on sensory neurons were indirect. For example, Chalazonitis et al. reported that TGF-β increased DRG neuron survival and substance P expression in vitro, but these effects depended on the presence of factors such as NGF and required non-neuronal support cells in the ganglion. In that study, neutralizing NGF with antibodies abolished TGF-β1’s neurotrophic effect, and TGF-β1 did not elevate NGF mRNA or protein levels, indicating that TGF-β1 was not acting directly on the neurons but rather through NGF. Furthermore, combining TGF-β1 and NGF did not produce an additive survival benefit. The authors speculated that TGF-β1’s neurotrophic activity is contingent on NGF or related molecules and works synergistically with NGF [12]. In contrast, our results clearly demonstrate that TGF-β1 acts directly on DRG neurons to produce significant effects without intermediate factors. This finding addresses the limitations of earlier work and confirms that TGF-β1 has a direct regulatory effect on sensory neurons.

It is important to note that our insights into these downstream changes come primarily from qPCR data rather than comprehensive profiling. In the future, transcriptomic sequencing and other high-throughput approaches will be needed to thoroughly map gene expression in DRG neurons under TGF-β1 treatment and to identify additional key factors involved in axon regeneration. Furthermore, functional studies are required to verify the roles of the identified factors (e.g., BMP4, NeuroD1, P21, osteopontin, TIMP1) in DRG regeneration and to clarify how they interact with the TGF-β/Smad3/4 axis. Concurrently, our findings indicate that in cisplatin-induced neurotrauma models, TGF-β fails to activate non-Smad signaling pathways such as MAPK, PI3K-Akt, or RhoA. Its regenerative effects are entirely dependent on the phosphorylation and nuclear translocation of Smad2/3, suggesting that the classical SMAD pathway represents the sole transmission channel for TGF-β-mediated nerve repair following cisplatin injury.

In CIPN, neurotrophic factors play a crucial role in facilitating axonal repair and regeneration. For example, cisplatin chemotherapy has been shown to downregulate endogenous BDNF levels, whereas exogenous BDNF supplementation can attenuate cisplatin’s neurotoxic effects [30]. Similarly, clinical studies have found an inverse correlation between NGF levels and CIPN severity—patients with greater NGF depletion tend to develop more severe neuropathy, implying that NGF deficiency exacerbates chemotherapy-induced nerve damage [31]. In vivo, glial cells (e.g., Schwann cells) respond to nerve injury by secreting neurotrophic factors such as NGF, BDNF, and glial cell line-derived neurotrophic factor (GDNF), along with anti-inflammatory cytokines like interleukin-10 (IL-10). This coordinated release creates a pro-regenerative microenvironment that promotes axonal regrowth and functional recovery [32]. We observed that glial cells and macrophages were virtually absent from the DRG cultures, and accordingly, qPCR analyses detected no significant changes in the neurotrophic factor or receptor expression levels over the course of the experiment. In addition, qPCR analysis revealed that TGF-β1 treatment and its receptor inhibitor SB431542 did not significantly change the expression of endogenous NGF or its receptor NGFR, suggesting that TGF-β1’s effect is not mediated by inducing the NGF/NGFR pathway. Further functional evidence supports the independence of TGF-β1’s action: co-treating DRG neurons with TGF-β1 and NGF did not produce any additive or synergistic effect on axon regeneration compared to either factor alone. These results indicate that TGF-β1 does not work indirectly by secondarily activating NGF or similar pathways but rather operates through an independent mechanism. Accordingly, in our cisplatin injury model, TGF-β1’s regenerative effect does not combine synergistically with NGF, and blocking TGF-β1 signaling does not weaken NGF’s effect. Beyond these experimental findings, significant hurdles remain before neurotrophic factors can be translated into effective therapies. These large protein molecules are susceptible to rapid degradation and do not readily cross physiological barriers such as the blood–nerve barrier, resulting in poor bioavailability at target peripheral nerves [33]. High doses are often required to achieve therapeutic effects, but dose escalation is constrained by safety concerns—for instance, early clinical trials of recombinant human NGF reported dose-limiting side effects, with injection-site hyperalgesia occurring in over 90% of treated patients [34]. Furthermore, delivering adequate concentrations of growth factors precisely to peripheral nerve injury sites remains challenging, underscoring the need for innovative delivery strategies to overcome barriers like the BNB. To surmount these obstacles, researchers are exploring approaches such as biomaterial-based delivery systems and gene therapy vectors that extend growth factor half-life and enable localized, sustained release at the injury site. These methods aim to raise the effective concentration of neurotrophic factors where they are needed while minimizing systemic exposure. Nevertheless, the aforementioned issues are not yet fully resolved, and thus neurotrophic factor-based therapies have so far seen only limited success in clinical applications for CIPN.

In summary, our study clearly demonstrates that TGF-β1 can act directly on DRG neurons and significantly promote axonal regeneration after cisplatin-induced injury via its downstream Smad3/Smad4 pathway. This pro-regenerative effect rarely relies on indirect mediators like NGF secreted by microglia or macrophage cells; instead, TGF-β1 engages an independent regenerative signaling network within DRG neurons. The ability of the TGF-β1/Smad4 axis to facilitate rapid recovery of damaged sensory neurons offers new insights and potential therapeutic targets for CIPN. Nonetheless, with a deeper understanding of TGF-β1’s role in peripheral nerve regeneration, new breakthroughs may be on the horizon, potentially leading to interventions that mitigate peripheral neurotoxicity in cancer patients.

## Figures and Tables

**Table 1:** Forward and reverse primers sequence of each gene analyzed by RT-qPCR.

Primer Name	Sequence (5′-3′)
18S rRNA	Forward:5′-CTGCCATTAAGGGCGTGGG-3′ Reverse: 5′-GTGATCACTCGCTCCACCTC-3′
B2M	Forward: 5′-ATCCAAATGCTGAAGAACGG-3′ Reverse: 5′-ATCAGTCTCAGTGGGGGTGA-3′
BMP4	Forward: 5′-GCCATTGTGCAGACCCTAGT-3′ Reverse: 5′-ACCCCTCTACCACCATCTCC-3′
NEUROD1	Forward: 5′-GCTGCGAGATCCCCATAGAC-3′ Reverse: 5′-ATGGCATTAAGCTGGGCACT-3′
N-CADHERIN	Forward: 5′-CACTGCCATTGATGCGGATG-3′ Reverse: 5′-TGCCACAGTGATGATGTCCC-3′
NGF	Forward: 5′-AAGTGCCGAGCCTCCAATCC-3′ Reverse: 5′-TTCTCATCTGTTGTCAACGCCTTG-3′
NGFR	Forward: 5′-CACAGCGACAGCGGCATCTC-3′ Reverse: 5′-AGCAGCTTCTCGACCTCCTCAC-3′
P21	Forward: 5′-TTGTCGCTGTCTTGCACTCT-3′ Reverse: 5′-AATCTGTCAGGCTGGTCTGC-3′
SMAD3	Forward: 5′-AGGGGCTCCCTCACGTTATC-3′ Reverse: 5′-CATGGCCCGTAATTCATGGTG-3′
SMAD4	Forward: 5′-TCAGCCAGCTACTTACCACCA-3′ Reverse: 5′-ACACGTCCCCTTCACCTTTAC-3′
SMAD7	Forward: 5′-TGCACAAAGTGTTCCCTGGT-3′ Reverse: 5′-AGCTGATCTGCACGGTGAAA-3′
SPP1	Forward: 5′-CTGCAGTTCTCCTGGCTGAA-3′ Reverse: 5′-TCTGGGTGCAGGCTGTAAAG-3′
STAT1	Forward: 5′-TTCAGCAGCTGGACTCCAAG-3′ Reverse: 5′-CGAGACATCATAGGCAGCGT-3′
TIMP1	Forward: 5′-TGTGCACAGTGTTTCCCTGT-3′ Reverse: 5′-TCTGGTAGTCCTCAGAGCCC-3′
TGFBR1	Forward: 5′-TGCTCCAAACCACAGAGTAGGC-3′ Reverse: 5′-CCCAGAACACTAAGCCCATTGC-3′
TGFBR2	Forward: 5′-CCTACTCTGTCTGTGGATGACC-3′ Reverse: 5′-GACATCCGTCTGCTTGAACGAC-3′
BDNF	Forward: 5′-TCGAAGAGCTGCTGGATGAGG-3′ Reverse: 5′-GGCTCCAAAGGCACTTGACTG-3′
TRKB	Forward: 5′-GGTCTATGCCGTGGTGGTGATTG-3′ Reverse: 5′-ACCGCCCTCCGAAGAAGATGG-3′
GDNF	Forward: 5′-CGCCGCCAATATGCCTGAA-3′ Reverse: 5′-TGCCGCTTGTTTATCTGGTGAC-3′
GFRα1	Forward: 5′-ACCACCACTGCCACGACTACC-3′ Reverse: 5′-GCACCAGCGAGACCATCCTTTC-3′

**Table 2:** siRNA sequence.

siRNA ^a^ Name	Sequence (5′-3′)
si-NC ^b^	Sense: 5′-UUCUCCGAACGUGUCACGUTT-3′
si-SMAD3 ^c^	Sense: 5′-UCCGGUUGACAUUGGACAGTT-3′
si-SMAD4 ^d^	Sense: 5′-UUGGAUUCUUUAAUAACAGTT-3′

^a^ Small interfering RNA; ^b^ nonspecific control; ^c^ Smad family member 3; ^d^ Smad family member 4.

## Data Availability

The original contributions presented in this study are included in the article/Supplementary Materials. Further inquiries can be directed to the corresponding authors.

## Supplementary Materials

The following supporting information can be downloaded at: https://www.mdpi.com/article/10.3390/cimb48040344/s1.
