# Supplementary material for: TGF-β1 Promotes the Recovery of Dorsal Root Ganglion Neurons from Cisplatin-Induced Injury Through Smad4-Dependent Mechanism
Source: Curr Issues Mol Biol. 2026 Mar 25;48(4):344. doi: 10.3390/cimb48040344 (PMC13115015; doi:10.3390/cimb48040344)

To further validate the expression changes of the significantly regulated genes, quantitative PCR was additionally performed using 18S rRNA as an alternative internal control. The results obtained using 18S rRNA were consistent with those normalized to β2-microglobulin (B2m), displaying similar expression trends across all experimental groups. This concordance supports the reliability of our qPCR data and confirms that the observed gene expression changes were not dependent on the choice of reference gene.

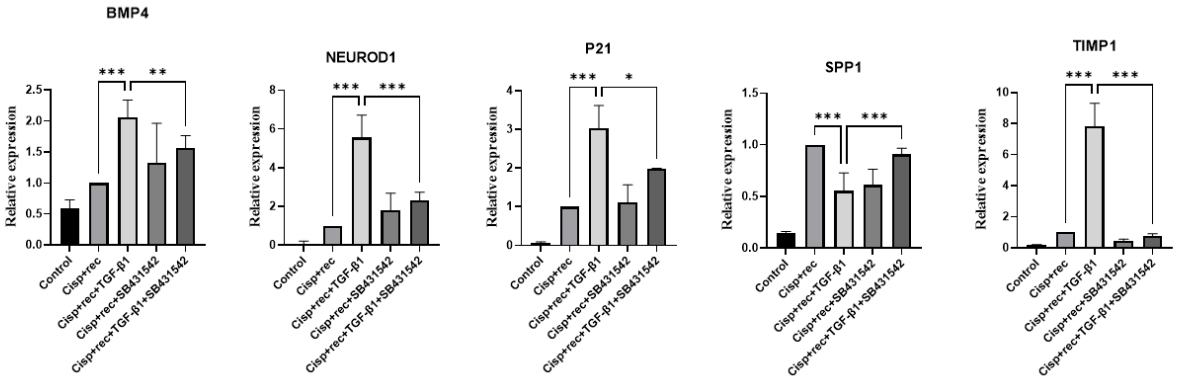

Supplement: Supplementary file 1 [file cimb-48-00344-s001.zip › Supplementary .pdf]
